# Origins and Consequences of Chromosomal Instability: From Cellular Adaptation to Genome Chaos-Mediated System Survival

**DOI:** 10.3390/genes11101162

**Published:** 2020-09-30

**Authors:** Christine J. Ye, Zachary Sharpe, Henry H. Heng

**Affiliations:** 1 The Division of Hematology/Oncology, Department of Internal Medicine, University of Michigan, Ann Arbor, MI 48109, USA; 2 Center for Molecular Medicine and Genomics, Wayne State University School of Medicine, Detroit, MI 48201, USA; zasharpe@umich.edu; 3 Department of Pathology, Wayne State University School of Medicine, Detroit, MI 48201, USA

**Keywords:** chromosome instability (CIN), fuzzy inheritance, genome theory, karyotype coding, two phases of cancer evolution

## Abstract

When discussing chromosomal instability, most of the literature focuses on the characterization of individual molecular mechanisms. These studies search for genomic and environmental causes and consequences of chromosomal instability in cancer, aiming to identify key triggering factors useful to control chromosomal instability and apply this knowledge in the clinic. Since cancer is a phenomenon of new system emergence from normal tissue driven by somatic evolution, such studies should be done in the context of new genome system emergence during evolution. In this perspective, both the origin and key outcome of chromosomal instability are examined using the genome theory of cancer evolution. Specifically, chromosomal instability was linked to a spectrum of genomic and non-genomic variants, from epigenetic alterations to drastic genome chaos. These highly diverse factors were then unified by the evolutionary mechanism of cancer. Following identification of the hidden link between cellular adaptation (positive and essential) and its trade-off (unavoidable and negative) of chromosomal instability, why chromosomal instability is the main player in the macro-cellular evolution of cancer is briefly discussed. Finally, new research directions are suggested, including searching for a common mechanism of evolutionary phase transition, establishing chromosomal instability as an evolutionary biomarker, validating the new two-phase evolutionary model of cancer, and applying such a model to improve clinical outcomes and to understand the genome-defined mechanism of organismal evolution.

## 1. Introduction

Chromosomal instability, or CIN, has increasingly become a topic of choice in cancer research. It also played an essential role in development of genome theory, a genomic theory of inheritance and evolution [1,2,3] (Box 1). There are many reasons why the importance of chromosomal instability becomes obvious within the current gene-centric world of molecular research: (1) chromosomal changes are overwhelmingly associated with cancers (both within the process of cancer evolution and as the end products) [4,5,6,7]; (2) karyotype coding, the spatial and topological coding of genes’ addresses within the nucleus, providing the physical platform of gene interaction networks, represents a new type of system inheritance which differs from gene-coded “parts inheritance” (thus it is the formation of a new karyotype that leads to the emergence of new genome systems) [8,9]; (3) macro-cellular evolution is driven by genome re-organization, while micro-cellular evolution is driven by gene mutation and/or epigenetic function [10,11]; (4) many previously characterized functions of cancer genes are in fact dependent on chromosomal instability or should be re-examined for the ignored involvement of CIN [12,13]; and (5) chromosomal instability can function as an effective biomarker with predictive and prognostic value in clinics, exceeding that of sequencing-based methodologies [14,15,16,17,18]. Perhaps one of the biggest hidden reasons for this shift is the research community’s response to the disappointing results of the gene-centric approach, which considers that cancer is a disease of gene mutations and promises to identify the few key common cancer gene mutations. Following nearly five decades of extensive research on cancer genes, especially current large-scale sequencing efforts to search for patterns of commonly shared gene mutations in different cancer types, the hugely divergent gene mutation profiles in all major cancer types forcefully challenge the theoretical basis of gene-based cancer research [19,20,21,22]. Studies that trace cancer evolutionary patterns using karyotype dynamics have linked some key pathways to the evolutionary mechanism of cancer [23]. Specifically, the vast majority of published cancer genes and molecular pathways can be traced back to, and unified by, chromosomal instability-mediated cancer evolution. The evolutionary mechanism of cancer can be expressed as the following:

### Evolutionary Mechanism = Collection of All Individual Molecular Mechanisms

Such a relationship suggests that each individual molecular mechanism has the potential to contribute to cancer under certain conditions as long as it can trigger the evolutionary mechanism of cancer and, perhaps more importantly, complete the long and difficult process of cancer evolution (from trigger factor to chromosomal instability to dominating population that breaks multiple levels of system constraints). Of course, unlike in experimental conditions, the majority of those cells initiated into cancer evolution will not be successful due to system constraints in vivo, such as immune surveillance, nutrient availability, and decreased fitness. Furthermore, in reality, no one single molecular mechanism can lead to cancer, as it usually involves a large number of combinations of individual mechanisms to advance different phases of evolution, a perfect storm of epigenetic–gene–genome–environment interactions where random factors make a significant contribution.

The understanding of evolution depends on analyses of “variable genotype and phenotype with differential fitness” and “evolving and constrained patterns of selection under different environmental pressures” [20,24,25,26]. Accordingly, the somatic evolutionary mechanism can be explained by four components. (1) stress, both initial internal and external; (2) genomic/epigenetic dynamics and cellular adaptation; (3) genome-based macro-evolution followed by gene/epigenetic-based microevolution; and (4) breaking the homeostasis of multi-level system constraints, e.g., normal cell regulation, cell populations, tissues, and organs. The four components work in the following order: cellular adaptation requires genetic and epigenetic changes and, as a trade-off, it leads to genome alterations. When the new genome becomes dominant, it can break the system constraints of the higher level. Even though only four key components are involved, stress and the stress response include many causative factors, including genomic and environmental factors, that are too numerous to be handled. Recently, a new cancer evolutionary model was proposed to reconcile the contribution from genome-mediated macro-evolution and gene-mediated micro-evolution: all individual molecular mechanisms can be classified as genomic/environmental interactions, which lead to macro-cellular evolution (the emergence of a new genome), followed by micro-cellular evolution to grow the cancer cell population and break the system constraints (tissues, organs) [3,27].

This new model has further explained some puzzling issues. For example, why it is much easier to link a gene mutation to cancer in experimental conditions. The aggressive molecular manipulations used in many experiments, such as the overexpression of cancer genes, can artificially promote cancer evolution in a linear model without essential heterogeneity and multiple levels of environmental constraints. In addition, researchers extensively select the phenotype of cellular growth and ignore the more important nature of the cancer, such as the instability of the cellular population and the emergence of a new system from it. Equally unfortunate is the study of cancer gene mutation without considering the phase of evolution (the trigger factors and the end products are not equal), and the general assumption that initial gene mutation occurs from a single cell which triggers somatic cell evolution.

Despite this understanding [12], the enthusiasm for the characterization of individual molecular mechanisms of CIN remains high. Surely, more links between CIN and other molecular mechanisms will be made under experimental conditions, including epigenetics, microRNA, non-coding RNA, micro-organismal interactions, metabolic profiles, and even lifestyle and social interactions. Due to this large number of possible contributing factors and their variance between individuals, focusing on each of them will be less useful in the clinic [2]. In contrast, in the context of cancer evolution, how to use CIN to predict clinical status is more important than identifying yet another molecular mechanism. To promote such a viewpoint and research priority, the importance of CIN (its origin and main function in cancer evolution) will be briefly reviewed, under the framework of how biosystems pass their information during somatic and organismal evolution, and the relationship between system behavior and the lower level agents that lead to its emergence. Because of the limitation on space for this essay, a few examples will be discussed, and more detailed discussions can be found from *Genome Chaos* [3]. This knowledge will shine new light on basic cancer research, as well as its implication for potential clinical usage.

Box 1 **Terminologies (modified from Horne et al., 2015 [28]. For more information, see Heng 2019 [3]).**

1. Genome theory of cancer evolution

The genome (a set of chromosomes for a given species) is a carrier of DNA as well as an organizer of genes. Specifically, the genome organizes the interactive relationship among genes through karyotype coding. The same or similar genes can form different genomes by re-organization of the genomic topology via karyotypic alterations, which are frequently observed in each evolutionary phase transition (transformation, metastasis, and drug resistance).

The relationship among gene mutations, epigenetic changes, and genome changes can be illustrated by the multiple-level landscape model where the local landscape represents gene/epigenetic status and the global landscape represents the status of genome replacement. Fundamentally, different bioprocesses require different types of inheritance, which should be studied in different landscapes. For example, genes/epigenetic mechanisms control developmental processes, while genome re-organizations are key for new system emergence.

Cancer evolution can be described as multiple runs of two-phase evolution. The key for cancer to become successful by overcoming all levels of constraint is to generate heterogeneity and differ from normal cells. This is most effectively accomplished through chromosome instability-mediated genome re-organization within the macro-evolutionary phase (including genome chaos). The gene mutation-mediated population growth will often follow within the micro-evolutionary phase. Currently, most sequenced gene mutation profiles are based on the end products of cancer evolution which differ from profiles detected from the cancer evolutionary process. Later-stage cancer gene mutations might make limited contributions to the macro-evolution of cancer.

2. Genome chaos (or karyotype chaos)

A process of massive and rapid genome re-organization following cellular crisis, which results in various chaotic genomes. For onvenience, the term “genome chaos” is also used to describe chaotic genomes (structural, numerical, and mixture types). This phenomenon was occasionally observed in cytogenetic studies, and it was largely ignored until the establishment of a link between it and the punctuated phase of cancer evolution [10]. In recent years, it was confirmed by sequencing across different cancer types, albeit with many different names (including “chromothripsis,” “chromoplexy,” “chromoanagenesis,” “chromoanasynthesis,” “chromosome catastrophes,” “structural mutations, ” “Frankenstein chromosomes” and more) [2,3,29,30]. Various molecular mechanisms, including telomere shortening-mediated crisis, can be linked to genome chaos [9,31]. Interestingly, different types of genome chaos are also observed from earlier development, though to a low degree and with low complexity. Genome chaos has been linked to high-stress conditions and is often observable during evolutionary phase transitions. Even though different studies have linked micronuclei, specific gene mutations, and various drug treatments to genome chaos, genome re-organization represents a general strategy for cellular survival by triggering the emergence of new genome systems. Genome chaos is a highly dynamic process, as a majority of the transitional chaotic genomes will not be detectable at the end of the process as only relatively more stable and simpler karyotypes will be selected. Genome chaos is a determinist process that creates new information systems with new phenotypes through both non-random and random action [32].

3. Fuzzy inheritance

To explain the limited predictability between genotype and phenotype and missing heritability, it was proposed that for a specific individual, each gene can code an array of phenotypes rather than two phenotypes being defined by classical binary categories (dominant vs. recessive). Environmental interaction functions as a selective window to generate a given phenotype within the coded potential range. In other words, from this “fuzzy” range of phenotypes, the respective environment can allow the best-suited status to be “chosen”. When passing inheritance to the next generation, it is the genomic package which codes an array of potential phenotype states, rather than the exact phenotype of parents [2,3].

4. Chromosome instability vs. genome instability

CIN is the rate (cell to cell variability) of changed karyotypes of a given cell population [12]. CIN includes structural CIN or numerical CIN. CIN can be measured by non-clonal chromosome aberrations, or NCCAs (both structural and numerical NCCAs). Genome instability is a broader concept, which includes CIN, copy number variations, DNA microsatellite instability (MSI), nucleotide instability (NIN), and CpG island methylator phenotypes (CIMPs). Note that clonal chromosome aberrations, or CCAs, should not be used to measure CIN. Initially, MSI and CIN were considered as mutually exclusive [33], but knowing the multiples types of genomic heterogeneity, a certain amount of overlapping between genetic and chromosomal instability is expected, especially under genome chaos.

5. Gene-centric framework vs. genome theory framework: answering the question of how biological inheritance works shapes genomic and evolutionary theories

Even though the genome theory perspective drastically differs from traditional concepts of chromosomes and evolution, it is less obvious to some who are only familiar with traditional concepts. For example, despite chromosome research having started in the early 1900s, and the importance of chromosomes in evolution having been suggested by Richard Goldschmidt and Barbara McClintock in the 1940s and Max King in 1990s [34,35,36], research interest in genes has overshadowed chromosomes, as there is no reason to prioritize chromosomal research within the gene era. Our recently introduced genome theory aims to change the status quo by providing the concept of karyotype coding. Similarly, proposing cancer as an evolutionary issue can be traced back to the 1970s [37], and cancer evolution is now a buzzword. However, the vast majority of cancer evolutionary studies are based on the neo-Darwinian principle, which cannot explain the phenomenon of rapid cancer evolution. Our two-phased cancer evolution discovery is still new for the majority of researchers. There are a number of important perspectives which can be used to distinguish between gene-centered and genome-based frameworks: a gene-centric framework considers chromosomes as the genes’ helpers; genes define inheritance; mutations are biological errors; the function of sexual reproduction is to mix genes for biodiversity; genes are the evolutionary selection unit; new genes define new species; the accumulation of gene mutations leads to cancer; micro-evolution over time leads to macro-evolution. In contrast, the genome theory framework states otherwise.

## 2. Stress as A Hidden Link: All Roads Lead to CIN

CIN studies used to be more straightforward. Most research targets are directly related to chromosomal machineries, including DNA replication/repair, chromatin condensation, condensed chromosome segregation, and regulators of cell cycle checking points. Traditionally, for example, gene mutations responsible for chromosome instability syndromes and drugs that can directly interfere with chromosomal machineries were frequently under investigation [38,39,40,41].

To explain the rapidly increasing linkages between diverse genes and cancer despite those genes not being directly related to chromosomal machineries, CIN has been classified into two types based on their trigger factors: For type I CIN, an individual contributing gene/pathway can directly impact chromosomal machineries to compromise genome integrity. Possible examples may include centromere alterations that result in mitotic chromosome mis-segregation. Similarly, the links between telomere and chromosomal behavior are well known, since the time of Barbara McClintock in the 1940s. In fact, many chromosomal instability syndromes belong to type I CIN. For type II CIN, individual contributing genes/pathways indirectly involve chromosomal machineries to jeopardize genome integrity through system response to general stress. The incidence of type II CIN (not directly related to chromosomal machinery) is far higher than that of type I, even though type I mechanisms have long been recognized. Interestingly, regardless of the trigger factors, internal genomic cause, external environmental cause, or classification into type I or type II, all categories are crucial for cancer evolution, and all can be measured by the degree of NCCA frequencies, as NCCAs represent a measurement of the baseline system instability [12].

**Figure 1:**
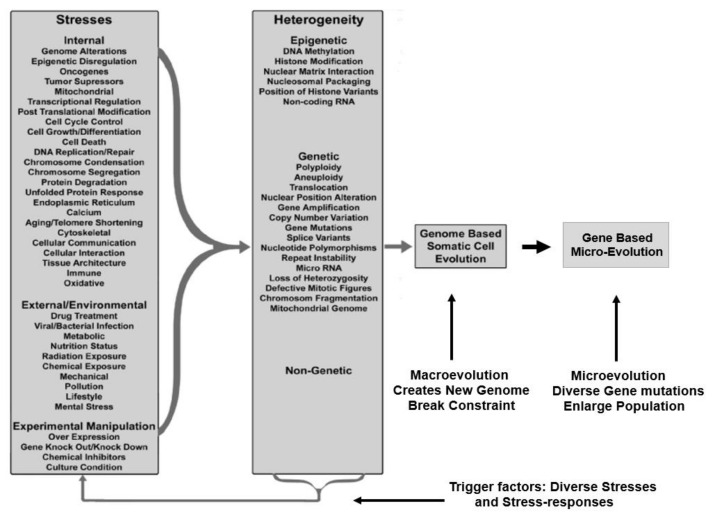
An illustration of the molecular mechanisms and their relationship with stress, genomic system response, macro- and micro-evolution, and genomic/environmental constraints. In the column of “Stresses”, each item, such as cell cycle control, could involve hundreds of gene mutations, and each mutation can represent a specific molecular mechanism. In the column “Heterogeneity”, different types of stress response can again involve vast amounts of different genomic and non-genomic factors or mechanisms. The types of genetic and non-genomic variations also include indels, duplications, and mobile elements [42]. Thus, the trigger factors are too numerous to handle. The key for cancer evolution can be understood as two main steps as soon as evolution is initiated: genome alteration-based macro-evolution followed by cancer gene mutations/epigenetic-based micro-evolution.

The classification of CIN reconciles why almost all pathways can be linked to chromosome instability and cancer evolution, which agrees with the evolutionary mechanism of cancer. Furthermore, it provides the conceptual framework to understand the hallmarks of cancer [28]. As illustrated in Figure 1, since there are so many different types of internal, external, and environmental stresses, including experimental stresses, many genes and pathways can be linked to cancer phenotypes, especially under ideal experimental conditions (which often represent some artificial linear models) [3]. However, for individual patients, the context of clinical reality makes knowledge derived from linear models less useful. Knowing this fact, researchers should no longer be keen on continuously characterizing individual molecular mechanisms, which may be virtually endless. The possible combinations of all types of internal and external stresses far exceeds our ability to investigate them all individually.

## 3. CIN: A Necessary Evil

Like discussions of stress, discussions of CIN often paint it as a solely harmful phenomenon. It is well accepted that both inherited gene mutations and many environmental insults (such as radiation, chemical treatment, infection by microorganisms) can lead to CIN [43]. In these cases, they are considered as a negative force. Recently, it is also realized that CIN is the trade-off of needed cellular adaptation [44]. To understand CIN, we will list some obvious reasons why CIN is unavoidable but sometimes also useful. In fact, CIN represents a system status, rather than simply being good or bad. CIN is context dependent [27].

First, many normal and essential bioprocesses have by-products which can damage biosystems, leading to CIN. Examples include the generation of reactive oxygen species from the process of energy production. Since it is impossible not to produce energy, CIN will always be with us. Additionally, stressors are omnipresent in the environment to varying degrees and can provide a trigger for an adaptive process via CIN.

Second, CIN is an evolutionary trade-off for essential cellular adaptation. In contrast to the traditional viewpoint, increased evidence supports the idea that CIN represents the price to pay for cellular adaptation. Since environments are highly dynamic, and the karyotype is fixed in the germline, the dynamic potential of CIN in somatic cells becomes important to allow chromosomal variants to occur for cellular adaptation. As a trade-off, the adaptation resulting from CIN can also contribute to disease potential [44,45]. This is related to the view of multicellular organisms as complex systems containing many individual components that display a range of potential activity and the ability to adapt to conditions as needed, instead of the identical and easily replaceable parts of a machine only able to function within limited tolerances. The adaptation/trade-off explanation shows cancer as an extreme consequence of this adaptability at the cellular level. There is a collaborative and conflicting relationship among different levels of biosystems, and cellular selection works on the current characteristics of the cell without regard for future problems for the higher levels of organisms. Fortunately, a small portion of individuals with cancer will not hugely impact on the species due to a separation of the germline and somatic genome [3].

This realization is of importance to answer the “why me” question for many patients. It also suggests that the goal of eliminating cancer in our generation is not realistic [2]. There are many examples of such a trade-off: a large portion of liver cells change their normal karyotype to polyploidy for the liver tissue’s function; antibody production can also introduce genome-level changes outside the antibody-specific region [46]; inflammation is necessary for response to injury and infection, but is also linked to chromosomal changes, including aneuploidy [47]. All of these trade-offs, reflected in genome-level alterations, contribute to and reflect CIN. The interesting twist is that, while CIN in somatic cells is “encouraged”, most CIN in germline cells is forcefully eliminated (under normal conditions) by the function of sexual reproduction [20,48,49]. Except under crisis conditions (e.g., massive extinction), when individuals with normal genomes no longer survive, they go through germline genome chaos to become different species with new re-organized genomes. In such situations, CIN likely becomes the key platform to re-organize the genome for new speciation events [1,3,20,50].

Third, the heterogeneity of each bioprocess can also generate different types of products, some of which are incomplete and can contribute to CIN. For example, incomplete cell death can lead to various chromosomal abnormalities, including chaotic genomes [51]. The heterogeneity of DNA repair and even the usage of RNA polymerase can favor CIN [41]. Of course, drastic environmental stresses can promote the heterogeneity of all bioprocesses, resulting in CIN. This explains why highly stressful environments (such as war conditions) and even lifestyles also impact the overall CIN [3,44,45,52].

Fourth, experimental manipulations can generate CIN through introducing stress to the system under investigation, which can often interfere with our data interpretation. However, most researchers are not aware of this important information. Many of the major molecular platforms for manipulating genomic systems, from transgenic vectors to small interfering RNA knockout and to CRISPR-Cas9 technologies, can generate significant levels of CIN and result in obvious system changes ([53] Heng, unpublished observations). Moreover, even experimental tools that do not direct the targeting of chromosomal machineries, such as specific molecular targeting and even cell culture conditions, can lead to CIN and new karyotypes. These observations are of importance, as when the karyotype changes, the expected molecular specificity no longer exists, as the genomic context is altered, resulting in changes in gene interaction networks. This is indeed the key reason why many molecular experiments cannot be repeated [3].

Fifth, the possibility exists that the good intention of medical treatment can paradoxically harm patients and may need to be separated from the above reasons due to its high significance. One example is that treatment-induced genome chaos can lead to massive and rapid drug resistance [2,3]. The rationale of using the maximum dosage of a drug treatment to kill as many cancer cells as possible seems obvious. However, the effective initial killing is often associated with rapid drug resistance. It is important to note that many common cancer therapies target genome repair processes and interfere with mitosis and cytokinesis, causing death in some members of a karyotypically heterogeneous tumor population, but inducing further CIN in outliers [48,54]. It is now gradually becoming understood that while aggressive treatment can kill more cancer cells, it can also induce genome chaos-mediated drug resistance. In the long term, the emergent resistance is much higher than it is with moderate treatment.

Lastly, CIN-mediated macro-evolution has played an important role in eukaryotic evolution, including human evolution [3]. It is not up to us to ask the evolutionary process to stop (even though combined technological evolution, one type of artificial evolution, could change the speed or even trajectories of human evolution). Many evolutionary processes are beyond our control (at least in current conditions). The evolutionary process got us where we are and will certainly be with us when we embrace the future. The rule of trade-offs is also applicable to technological evolution, where we hope to achieve maximal benefit at minimal cost.

Another interesting phenomenon is that under crisis conditions, the survival strategy for cancer genomes is to create new genomes by “sacrificing” their own, as genome chaos leads to massive re-organization, and afterwards the surviving genomes are no longer the same compared with the populations prior to treatment. This mechanism supports the natural genome engineering concept, which is important for organismal evolution [2,3,20,55,56,57].

In summary, CIN is unavoidable for advanced life systems both from internal sources (fuzzy inheritance seems to have been favored by evolution for evaluability) and environmental challenges [3]. It is essential for cellular adaptation as it can be linked to arrays of non-genomic and genomic variations, including epigenetic changes, gene mutations, copy number variations, genomic transfer among cells, chromosomal aberrations and genome chaos, and somatic mosaicism, involving many biological processes [13,46,58,59,60,61,62,63,64]. Among them, some non-genomic changes, including epigenetic variations, represent a lesser degree of impact in terms of inducing CIN, and genome chaos represents an extremely high degree of impact. When applying such realizations to organismal evolution, CIN maintenance and creation at the germline level becomes a powerful mechanism for organismal evolution, especially during massive extinctions [3].

## 4. CIN is Essential for Emergence of New Cellular Populations

Despite the “out of control growth” phenotype, cancer, fundamentally, is a phenomenon of a new cell population with an altered genome emerging from normal tissue. Comparable to the observation that the vast majority of animal and plant species display different karyotypes, cancer could be considered as a different emergent species or cellular species [2,3,20,50,65,66,67]. Such a mindset is also helpful to understand why CIN plays a dominant role in cancer evolution. For example, if only viewed from a genomic “parts” or agent point of view, it is confusing as to why so many genes (agents) can be linked to cancer in experimental models yet the clinical prediction of these genes in cancer is low. From a system behavior point of view, however, it becomes obvious that there is no linear relationship between lower level agents and the behavior of higher level systems. Moreover, from an evolutionary point of view, the cellular population transition is driven by CIN which can be contributed to by a large number of agents. Within the macro-cellular phase, the evolutionary meaning of all lower level agents (genes, epigenetic function, or pathways) can only be “visible” when they have impacted on CIN. That is why cancer research should not focus on the lower levels of the system’s agents, as even though different lower level agents can be linked to cancer, their individual contribution is limited. Environments above the individual cell, such as tissue or organ organization, and other even higher systems, can all be considered as evolutionary constraints for cellular populations. No wonder overwhelming chromosomal changes have been detected from a majority of cancers by genome sequencing, and when chronic myeloid leukemia (CML) enters the chromosomal dynamics phase (blast crisis phase), the “magical” drug of imatinib is no longer so [68,69]. It is known that a majority of solid cancer cells display genome-level instability and a much smaller portion display microsatellite instability, and even microsatellite instability can contribute to CIN. Furthermore, caution is needed during cancer treatment when such treatment can induce CIN, possibly triggering genome chaos, with unpredictable consequences.

Interestingly, the importance of CIN and its relationship with genes in cancer also promotes the concept that when there are multiple levels or scales involved, it is useful to identify the hierarchy of the levels of study, as well as best technical platforms to focus on this [1]. Similarly, we need to choose different technical platforms for a specific biological question, by weighting different contributions of genomic and non-genomic variants, including massive chromosomal alterations, simple chromosomal changes, copy number variations, nucleotide polymorphisms, and epigenetic alterations. For example, if it is a developmental issue, profiling the time and space of gene expression is effective. If, however, it is a macro-evolutionary issue, tracing karyotype evolution makes much more sense than studying an individual gene’s function. When making such a choice, the phase of evolution, the status of the average and outliers of population [54], the stability of the higher level constraints, and the degree of environmental stress need to be carefully considered [3].

## 5. Conclusions and Future Perspective

While the goal of molecular cancer research is to identify targets for cancer diagnosis and treatment, the challenge has become daunting as increased knowledge of molecular mechanisms has failed to apply to the clinic. At the early stage of cancer research, it is critical to understand the molecular pathways that directly or indirectly drive CIN. Following efforts over several decades, many links have been identified as triggering factors and consequences of CIN. The same effort will certainly last years, if not decades. However, knowing that cancer evolution is not linear, and that CIN-mediated macro-evolution is highly unpredictable based on trigger factors, efforts should focus on how to apply evolutionary principles to treat cancers. The following future perspectives provide some rationales for studying CIN and its implications via an evolutionary lens.

(1) CIN research should not continuously focus on the characterization of additional molecular pathways. As illustrated in Figure 1, there should be many more individual mechanisms of CIN which can be studied. Even though these mechanisms can be linked to the hallmarks of cancer, the clinical significance is limited [28], as cancer, like many common and complex diseases, is not simply a polygenetic disease [70]. In contrast, we need to focus on searching for new platforms of studying genome-defined, network–environment interaction-mediated, evolutionary phase transitions of cellular populations. To illustrate how network dynamics promote or constrain cancer evolution, the details of how karyotype coding defines network structure and regulates environmental stress are key [1,3,8].

(2) Establishing CIN as an evolutionary biomarker: as cancer is an evolutionary process, a better biomarker to monitor the evolutionary potential, which can be used to predict the evolutionary trajectory, is a critical need [2,3,10,11]. Such a biomarker should be able to pinpoint the phase of evolution so that different research or treatment platforms can be applied accordingly. To achieve this goal, further study of the types of NCCAs is needed to record them and to understand the relationship among different types of NCCAs [13,27]. In addition to structural and numerical chromosome changes, many unclassified chromosome and nuclear structures, including micronuclei clusters, need to be included [8,9]. Micronuclei clusters are a group of smaller nuclei with variable sizes. They can be formed from either diploid or polyploid cells, being a type of NCCA. Recent work has shown a critical link between micronuclei formation and massive genome rearrangements [29], along with cancer progression and metastasis [71]. In this regard, the micronucleus not only provides a means of adaptation via large-scale genome rearrangement, resulting in a more stable clonal chromosomal aberration, but also a way of escaping the system constraints imposed on the tumor by the surrounding tissue. In addition, the complexity of the structural changes also need to be studied. The goal is to establish a combined biomarker which can have quantitative value.

(3) Monitoring treatment using CIN to reduce induced genome chaos-mediated drug resistance: the appreciation of cancer as an evolution process demands a new strategy for cancer treatment. For example, one general strategy is to kill cancer cells using maximal treatment power. Knowing that the massive killing power can also trigger genome chaos-mediated rapid drug resistance and result in more aggressive cancer cells, a more systematic comparison needs to be done for currently used treatments. Recently, an approach termed adaptive therapy has received increased attention [72,73]. By considering the evolutionary behavior of drug-sensitive and -insensitive cancer cells, such treatment might mainly reduce the induction of genome chaos and target the clonal population at the micro-evolutionary phase. Based on this concept, moderately targeting cells with specific cancer gene mutations should spare certain portions of the dominant cancer population compared to trying to kill most of them. As long as massive genome chaos can be avoided, the cell population can be constrained without creating new genomes.

It should be noted that many current therapeutic strategies are based on the rationale of inducing excessive levels of CIN that are not compatible with viability [74,75,76,77,78]. Despite the effective killing, the emergence of drug resistance is common. It is most likely that induced genome chaos plays a key role in drug resistance caused by targeting CIN. Since cancer macro-evolution is a game of outliers [2,3], the cancer cell killing strategy based on the average cell profile needs rethinking.

(4) Validating the new evolutionary model of cancer: based on the importance of CIN in macro-evolution and cancer gene in micro-evolution, a new cancer evolutionary model was proposed [3,27]. This model places macro-cellular evolution (where genome re-organization dominates) prior to micro-cellular evolution (where cancer gene mutation promotes population growth) and emphasizes the importance of CIN for generating new cancer genomes. This model fits well with both karyotype data and sequencing data from various cancer models [3,10,21,71,79,80,81,82,83]. More validation experiments are needed to examine this model.

(5) Applying somatic evolutionary information to understand organismal evolution: studies of cancer evolution have unexpectedly provided much insight into organismal evolution [1,2,3]. First, the two phases of somatic evolution are separated into macro- and micro-evolution, with two distinctive forms of genomic information systems. This differs from the current organismal evolution concept where macro-evolution equals the accumulation of micro-evolution over time. Second, during the micro-evolution phase, animals’ and plants’ germline genomes are very stable, differing from somatic cells that display certain baseline levels of CIN. Interestingly, during the crisis stage (e.g., massive extinction), a majority of species are wiped out (small portions of species can survive due to the survivability of their own “lucky” genomes). Many new types of species will finally emerge following massive genome re-organization induced by massive extinction, plus increased opportunities to allow individuals with similar altered genomes to reproduce. In this phase, CIN becomes the key strategy to pass the torch of life, albeit through different species.
